# Generating Chimeric Zebrafish Embryos by Transplantation

**DOI:** 10.3791/1394

**Published:** 2009-07-17

**Authors:** Hilary A. Kemp, Amanda Carmany-Rampey, Cecilia Moens

**Affiliations:** HHMI and Division of Basic Sciences, Fred Hutchinson Cancer Research Center - FHCRC

## Abstract

One of the most powerful tools used to gain insight into complex developmental processes is the analysis of chimeric embryos. A chimera is defined as an organism that contains cells from more than one animal; mosaics are one type of chimera in which cells from more than one genotype are mixed, usually wild-type and mutant. In the zebrafish, chimeras can be readily made by transplantation of cells from a donor embryo into a host embryo at the appropriate embryonic stage. Labeled donor cells are generated by injection of a lineage marker, such as a fluorescent dye, into the one-cell stage embryo. Labeled donor cells are removed from donor embryos and introduced into unlabeled host embryos using an oil-controlled glass pipette mounted on either a compound or dissecting microscope. Donor cells can in some cases be targeted to a specific region or tissue of the developing blastula or gastrula stage host embryo by choosing a transplantation site in the host embryo based on well-established fate maps.

**Figure:**
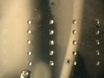


## Protocol

A step-by-step guide to generating targeted chimeric zebrafish embryos by transplantation at the blastula or gastrula stage.

One of the most powerful tools used to gain insight into complex developmental processes is the analysis of chimeric embryos. A chimera is defined as an organism that contains cells from more than one animal; mosaics are one type of chimera in which cells from more than one genotype are mixed, usually wild-type and mutant. In the zebrafish, chimeras can be readily made by transplantation of cells from a donor embryo into a host embryo at the appropriate embryonic stage. Labeled donor cells are generated by injection of a lineage marker, such as a fluorescent dye, into the one-cell stage embryo. Labeled donor cells are removed from donor embryos and introduced into unlabeled host embryos using an oil-controlled glass pipette mounted on either a compound or dissecting microscope. Donor cells can in some cases be targeted to a specific region or tissue of the developing blastula or gastrula stage host embryo by choosing a transplantation site in the host embryo based on well-established fate maps.

### Part 1: Injecting Zebrafish Embryos at the 1-cell stage.

**Enzymatic dechorionation of 1-cell stage embryos.** To proteolytically dechorionate embryos, incubate at the 1-cell stage for ~1-5’ in a 0.5 mg/ml pronase solution as described in the Zebrafish Book 1. Moniter dechorionation closely and submerge embryos in Embryo Medium (EM) with Pen/Strep 1 as soon as the chorions begin to visibly collapse. Once released from their chorions, blastula and gastrula stage embryos are fragile and will stick to plastic or disintegrate if exposed to air. Therefore keep dechorionated embryos submerged in Pen/Strep EM, transfer between dishes using a wide-bore fire-polished glass pipet, and maintain in either agar-coated plastic dishes (1.2% agarose in Pen/Strep EM) or autoclaved glass petri dishes.
**Injecting dechorionated embryos with lineage marker.** Prepare an injection dish by inserting a glass slide at a 45 degree angle across the widest part of a Petri dish three-quarters full of 1.2% agarose made in Pen/Strep EM. Removal of the slide after agarose solidification leaves a beveled trough. Transfer dechorionated embryos into the trough of an injection dish filled with Pen/Strep EM. Inject 1nl volumes of lineage marker using a precisely calibrated fire-pulled glass injection pipette and a pressure injection rig, as described in the Zebrafish Book 1. Inject dyes and low molecular-weight lineage tracers directly into the yolk of dechorionated embryos between the 1- and 4-cell stages. A 3% solution of fluorescent dextran is sufficient to allow for the detection of donor-derived cells in chimeric embryos through several days of development. When the lineage marker is an mRNA encoding a fluorescent protein (for instance, GFP or RFP), inject directly into the cell of the early 1-cell stage embryo.
**Injecting non-dechorionated embryos with lineage marker.** Prepare multi-well injection dishes by solidifying 1.2% agarose in EM around a mold with wells roughly the same width as the chorion diameter. Transfer non-dechorionated embryos into a multi-well injection dish half-filled with Pen/Strep EM. Remove excess fluid. Thus immobilized, embryos can be injected with 1nl volumes of lineage marker as described above.
**Raising injected embryos for transplantation.** Raise embryos at low density (40-50 embryos per dish) in fresh Pen/Strep EM. Stage-match the donor and host embryos used for transplantation fairly closely. For shield stage transplants, aim for the donors to slightly lag behind the hosts; note that injected embryos tend to be slightly delayed. Incubate embryos at different temperatures, 25°C, 28°C, and 31°C, to stagger their development and maximize the time window over which transplants can be performed.

### Part 2: Making chimeric zebrafish embryos by transplantation.

**Transplantation at blastula stages on the stereomicroscope**

**Description of the transplant rig for blastula** The apparatus used for cell transplantation in the zebrafish blastula consists of a micrometer drive-controlled Hamilton syringe (10  l-50 l) attached by a three-way stopcock to a reservoir of mineral oil and to a micropipette holder through a length of flexible tubing. After assembling the transplantation rig, it is filled with mineral oil, taking care to eliminate all air bubbles from the system. The presence of an air bubble will negatively impact your ability to control the suction and pressure. Use a stereomicroscope with fairly high magnification and good optics (ideally at least 80x magnification). The positioning of the micropipette holder and needle can be controlled by a Narishige manual micromanipulator, as for injections; however, if the base of the stereoscope is too wide to allow easy access with the micromanipulator, then an adaptor that attaches to the body of the stereomicroscope can also be purchased from Narishige. The mounting of the micromanipulator must be very stable in order to avoid transferring vibrations to the transplant needle; a magnetic base works well for this purpose.
**Preparing the transplantation pipette** Transplantation needles are made from glass capillary pipettes (see two options in reagents table below) that are drawn to a gentle taper on an electrode puller. Break the tip of needle off under a dissecting microscope using a straight edge razorblade at the point where the inner diameter of the needle is slightly larger than the cells to be transplanted. Hold the razorblade at a slight angle to create a bevel and make the break as smooth as possible, with no jagged edges. For blastula stage transplants the outer diameter of the needle should measure approximately 50-60 mm.
**Mounting embryos for transplantation in agar molds** Prepare injection dishes by floating a plastic mold with rows of wedge shaped protrusions in a Petri dish that is half-filled with molten 1.2% agarose in EM. Once the agarose has solidified, remove the template, leaving an agar mold that contains rows of triangularly shaped wells each just large enough to hold one embryo. Fill the transplantation mold with Pen/Strep EM and load blastula stage embryos individually into each well with a fire polished glass pipette so that the donor embryos are placed down one column and the host embryos are placed down the adjacent column.
**Transplanting cells** Position embryos in the transplant wells on their sides; reposition with the transplant pipette as needed during transplantation, taking care not puncture the yolk. Position the dish on the stage so that when the transplantation needle enters an embryo, the needle pushes the embryo against the back wall of its well. Using the micromanipulator, lower the transplantation needle into the dish at a fairly steep angle. Ideally the transplantation needle should enter the embryo at approximately a 45-degree angle. Once the tip of the needle is below the surface of the embryo medium, draw a small amount of embryo medium into the needle by twisting the micrometer drive controlling the Hamilton syringe. For the most precise control, the interface between the mineral oil and the embryo medium should remain in the thin, tapered part of the needle, but not too close to the end. Gently position the donor embryo with the needle and then draw the needle back and enter the blastula cap of the embryo at the desired position. A swift, hard hit will penetrate the embryo without causing it to roll. Draw up donor cells slowly and carefully into the needle. If the cells are taken up too quickly, they are likely to shear. Also avoid taking yolk up into the needle, as it will bind to the mineral oil, which will then kill the cells. After the desired number of cells is taken up, reverse the pressure slightly to stop the suction and remove the needle from the embryo. Bring the host embryo into position by moving the transplantation dish. Small adjustments to the position of the host embryo can be made by gently turning it using the transplantation needle, as long as care is taken to not touch the yolk. When expelling the donor cells into the host embryo, avoid introducing a large amount of embryo medium or any mineral oil, as this could interfere with development or kill the embryo. The position of cells along the animal-vegetal axis influences their contribution to one of the three germ layers, endoderm, mesoderm, and ectoderm 2,3 . Accordingly, cells transplanted close to the margin prior to the beginning of gastrulation will give rise preferentially to endoderm and mesoderm, while cells transplanted toward the animal pole will contribute to ectodermal fates, most commonly surface ectoderm, forebrain and eyes. Thus a degree of tissue targeting can be accomplished even at blastula stages. After the cell transfers have been completed, transfer the donor-host pairs to the agar coated wells of a 24 well plate to develop further.

**Transplantation at gastrula stages on the compound microscope**

**Description of the transplant rig** Cell transfers performed on a compound microscope benefit from the precise control of needle position provided by a three-axis oil hydraulic micromanipulator. This micromanipulator controls the fine movements of the transplant pipette, while the suction in the pipette is controlled by a micrometer-drive Hamilton syringe similar to the one used for blastula transplants. An upright compound microscope with a fixed stage is preferable for performing cell transfers since focusing such a microscope does not cause the embryo to move relative to the pipette. However, if an adjustable-stage compound microscope must be used, then the micromanipulator can be mounted to the stage rather than to the body of the microscope with the same effect. Adaptors that provide options for mounting the micromanipulator to either the stage or body of compound microscopes from a variety of manufacturers are also available from Narishige.
**Preparing the transplantation pipette** Transplantation needles are made essentially as described above; for gastrula stage transplants the ideal needle aperture is 30-40 mm. After shield formation, the enveloping layer (EVL) becomes more adherent, making it difficult for the transplantation needle to penetrate the embryo. To facilitate needle entry, pull a barb on the end of the needle using a microforge. First, smooth the tip of the needle by bringing it close to the filament of the microforge; then turn the needle so that the leading edge of the bevel can make contact with the filament. Once the leading edge of the needle contacts the filament, pull it quickly away to create a barb, or harpoon. To avoid damaging cells, the barb should be straight and the tip of the needle should not curve
**Mounting embryos for transplantation in methylcellulose** For compound microscope transplants, mount embryos on a depression slide in a 3% solution of methylcellulose (Sigma) dissolved in embryo medium. First, smear a vertical stripe of methylcellulose in the depression of a glass depression slide, then flood with a generous amount of embryo medium. Using a fire-polished pipet, transfer one donor and three shield-stage hosts under the surface of the EM on the depression slide on one side of the methylcellulose. Choose donor embryos that are slightly younger than the hosts (30-50% epiboly). With a small loop made by gluing the ends of a short length of 2-lb test fishing line into the end of a capillary tube, gently roll the donor and host embryos up onto the top of the methylcellulose strip and stuff them down into the methylcellulose until secure. If the transplanted cells are to be targeted to a particular domain, knowledge of the shield stage fate map 3,4  and the position of the target tissue relative to the shield is essential so that host embryos can be positioned correctly. As the host embryos are placed into the methylcellulose, roll them into position so that the targeted region is uppermost, since during transplantation the needle will enter the embryo tangentially to deliver the donor cells without damaging the yolk.
**Transplanting cells** Position the transplantation needle by moving it into the focal plane of the embryo. First focus on the donor embryo using the 10x objective. Then move the embryo off to the side, and without adjusting the focal plane, use the controls of the micromanipulator to bring the tip of the transplantation needle into focus. Position the micropipette holder and needle at a fairly shallow angle, so that the needle is as close to horizontal as the edge of the depression on the slide will allow. If an embryo is mounted properly in methylcellulose, it will not roll as the transplantation needle enters, unless the embryo is too old to allow the needle to penetrate easily. The enveloping layer seems to toughen as embryos age, therefore, gastrula stage transplants are best performed right after shield formation. After dome stage, the yolk resides just under the cells of the blastoderm cap, which progressively thins as epiboly proceeds. In order to avoid piercing the yolk, the needle should enter the embryo at a focal plane that is just slightly deeper than that of the EVL. When removing large numbers of cells from the donor embryo, move the needle around as the cells are taken up, in order to avoid accidentally sucking yolk into the needle. If cells from one donor embryo are to be transplanted to multiple hosts, it is best to enter the donor with the needle only once in order to minimize the possibility of damage. The donor cells can then be transferred to the desired location in up to three host embryos. When transferring cells from two or more donor embryos into one host embryo, it is best to take cells from each of the donor embryos into the same transplantation needle before transplanting them to a host embryo. This minimizes damage to the host embryo, and ensures that the cells are transferred to the same area of the embryo, so that their behaviors can be compared. Very little mixing occurs between the donor cells within the transplant needle, therefore, the cells should be transferred to a single host when more than one donor is used. In order to increase the likelihood that the donor cells will contribute to the desired tissue, expel the donor cells while the needle is being drawn through the targeted area, as opposed to depositing the cells in a single clump. Once the cell transfers are completed, place the entire slide into a petri dish and flood carefully with Pen/Strep EM. Over the next few hours the methylcellulose will dissolve, releasing the embryos.

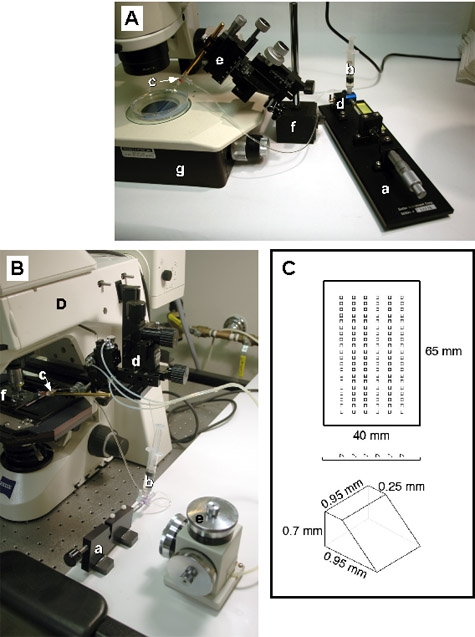
 **Figure 1:**Microscope set-up for making chimeric embryos. A: A dissecting microscope transplant rig consists of an oil-filled Hamilton syringe with a micrometer drive (a) connected to an oil-filled reservoir (b) and the transplant pipette (c) via a 3-way stopcock (d). The transplant pipette is mounted on a coarse micromanipulator (e) which is attached to a metal base plate (not shown) via a magnetic foot (f). Transplants are performed on the stage of a stereomicroscope (g) equipped with bottom lighting for optimal optics. B: a compound microscope transplant rig consists of a similar oil-filled Hamilton syringe and micrometer drive (a,b) but in this case the transplant pipette is mounted on a pipette holder (c) whose X, Y and Z movements can be controlled either by a coarse (d) or fine (e) micromanipulator. In this example, the micromanipulator is mounted on the body of a fixed-stage microscope, however it is also possible to mount it on the stage of a regular microscope. The embryos, which are immobilized in methylcellulose on a depression slide, are visualized using a 10x objective (f). C: Transplant mold for immobilizing embryos for stereomicroscope transplants. Dechorionated embryos are dropped individually into wells made by casting this mold into agarose in a 90mm Petri dish. The dimensions of the wells are shown.


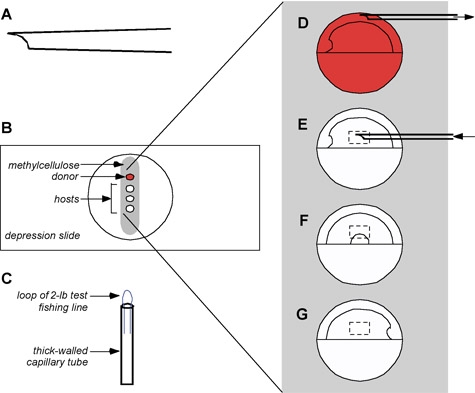
 **Figure 2:**Mounting embryos for gastrula-stage transplants. A: ideal shape of a gastrula transplant pipette. A bevel with a sharp tip aids in penetrating the embryo. B: Immobilizing donor and host embryos in methylcellulose. A strip of methylcellulose is laid down in the well of a depression slide and flooded with a generous amount of embryo medium. Embryos are added to the embryo medium and are rolled onto the methylcellulose using a small loop (C). D-G enlargement of embryos in Fig. 1B. D: The donor embryo is oriented to allow easiest access for the pipette, since cells at this stage are still uncommitted. E-G: Shield-stage host embryos are oriented so that the target region is uppermost. Cells transplanted into the boxed regions will contribute to dorsal hindbrain and cranial neural crest derived from the left (E) and right (G) sides or to the ventral hindbrain and spinal cord (F).


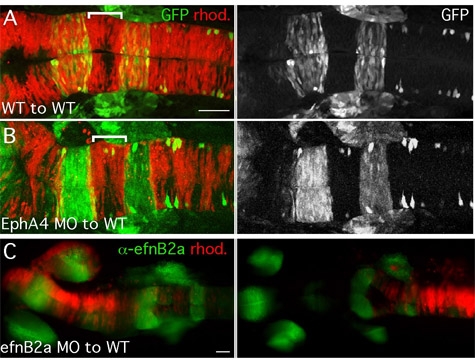
 **Figure 3:** Representative images of chimeric embryos. (A-C) 18hpf chimeric embryos generated by transplantation at shield stage shown in dorsal view, anterior to the left. (A,B) Sorting functions for cell surface receptor EphA4 revealed by analysis of chimeric embryos.  Left panels: merge of rhodamine-labeled (rhod.) donor cells (red) and transgenic GFP expressed in specific hindbrain segments (green). (A) WT cells contribute to the entire hindbrain of a control chimeric embryo.  (B) EphA4-depleted donor cells are excluded from specific segments in the hindbrain of a WT host. (C) Donor cells targeted to different parts of the neural tube in shield-stage transplants.  Donor cells (red) targeted to the forebrain/midbrain (left panel) or hindbrain/spinal cord (right panel) of a WT host counter-stained with an EfnB2a antibody that marks the forebrain, mid-hindbrain boundary and the middle segment of the hindbrain (green). Scale bars: 50μm.

## Discussion

The ease with which transplantation can be used to produce targeted chimeras is one of the great powers of the zebrafish as a vertebrate model. A modification of this protocol not described above allows analysis of maternal gene function for genes with essential roles in zygotic development. Since these mutant fish cannot survive until adulthood, it is necessary to transfer the mutant germline into an otherwise wild-type host embryo, creating “germline clones” of mutant cells. Generating germline mosaics involves transplanting primordial germ cells from a mutant donor to a wild-type host embryo. Unlike all other cell lineages in the embryo, the germ cell lineage is specified very early in development by the inheritance of maternal determinants ^5,6^ . Thus, the first challenge of making germline mosaics is identifying the primordial germ cells (PGCs) in the donor embryo. At midblastula stages the PGCs reside along the margin ^7,8^ , so picking up 50-100 random cells from the margin of a lineage-labeled donor embryo frequently results in the transfer of PGCs. When transferred to the animal pole of an unlabeled host embryo, the primordial germ cells actively migrate towards the presumptive gonad where they can be unambiguously identified at 24 hours of development by their characteristic position, large size, and dye retention due to their slow proliferative rate ^9-11^ . Meanwhile, donor-derived non-PGCs will acquire the fate determined by their location in the host: primarily forebrain and eyes if they were transplanted to the blastula animal pole. Injecting the host embryo with morpholinos that knock down the *dead end* gene effectively eliminate the host germline in a cell-autonomous manner, so that even a single donor-derived PGC can repopulate the entire germline ^10,12^ (C.M., personal observation). More recently, a transgenic line that allows PGCs to be visualized in live embryos during blastula stages has been developed ^13^. When crossed into the mutant whose maternal function is to be determined, this transgene, combined with the *dead end* morpholinos, will make germline transplants facile because single PGCs can be identified and transplanted into a germline-depleted host.
